# COVID-19 vaccine trials: The use of active controls and non-inferiority studies

**DOI:** 10.1177/1740774520988244

**Published:** 2021-02-03

**Authors:** Thomas R Fleming, Philip R Krause, Martha Nason, Ira M Longini, Ana-Maria M Henao-Restrepo

**Affiliations:** 1 Department of Biostatistics, University of Washington, Seattle, WA, USA; 2 Office of Vaccines Research and Review, FDA/CBER, Silver Spring, MD, USA; 3 National Institute of Allergy and Infectious Diseases (NIAID/NIH), Bethesda, MD, USA; 4 Department of Biostatistics, University of Florida, Gainesville, FL, USA; 5 World Health Organization, Geneva, Switzerland

**Keywords:** COVID-19, SARS-CoV-2, vaccines, placebo-controlled, active comparator, non-inferiority, margin

## Abstract

**Background:**

Recently emerging results from a few placebo-controlled randomized trials of COVID-19 vaccines revealed estimates of 62%–95% relative reductions in risk of virologically confirmed symptomatic COVID-19 disease, over approximately 2-month average follow-up period. Additional safe and effective COVID-19 vaccines are needed in a timely manner to adequately address the pandemic on an international scale. Such safe and effective vaccines would be especially appealing for international deployment if they also have favorable stability, supply, and potential for implementation in mass vaccination campaigns. Randomized trials provide particularly reliable insights about vaccine efficacy and safety. While enhanced efficiency and interpretability can be obtained from placebo-controlled trials, in settings where their conduct is no longer possible, randomized non-inferiority trials may enable obtaining reliable evaluations of experimental vaccines through direct comparison with active comparator vaccines established to have worthwhile efficacy.

**Methods:**

The usual objective of non-inferiority trials is to reliably assess whether the efficacy of an experimental vaccine is not unacceptably worse than that of an active control vaccine previously established to be effective, likely in a placebo-controlled trial. This is formally achieved by ruling out a non-inferiority margin identified to be the minimum threshold for what would constitute an unacceptable loss of efficacy. This article not only investigates non-inferiority margins, denoted by **δ**, that address the usual objective of determining whether the experimental vaccine is “at least similarly effective to” the active comparator vaccine in the non-inferiority trial, but also develops non-inferiority margins, denoted by **δ_o_**, intended to address the worldwide need for multiple safe and effective vaccines by satisfying the less stringent requirement that the experimental vaccine be “at least similarly effective to” an active comparator vaccine having efficacy that satisfies the widely accepted World Health Organization–Food and Drug Administration criteria for “worthwhile” vaccine efficacy.

**Results:**

Using the margin **δ** enables non-inferiority trials to reliably evaluate experimental vaccines that truly are similarly effective to an active comparator vaccine having any level of “worthwhile” efficacy. When active comparator vaccines have efficacy in the range of 50%–70%, non-inferiority trials designed to use the margin **δ_o_** have appealing properties, especially for experimental vaccines having true efficacy of approximately 60%.

**Conclusion:**

Non-inferiority trials using the proposed margins may enable reliable randomized evaluations of efficacy and safety of experimental COVID-19 vaccines. Such trials often require approximately two- to three-fold the person-years follow-up than a placebo-controlled trial. This could be achieved, without substantive increases in sample size, by increasing the average duration of follow-up from 2 months to approximately 4–6 months, assuming efficacy of the active comparator vaccine has been reliably evaluated over that longer duration.

## Introduction

Safe and effective vaccines that meaningfully reduce the spread of SARS-CoV-2 virus will have indisputable value in addressing the COVID-19 pandemic, which has disrupted health and taken lives around the world. New vaccines have been developed and testing begun at an unprecedented pace, with at least seven vaccines in ongoing placebo-controlled randomized trials.^1^ Additional vaccines are expected to enter placebo-controlled trials soon, including through the imminent initiation of the World Health Organization (WHO) Solidarity Vaccines Trial^2^ that follows the principles of a core protocol.^3^ This platform trial is designed to evaluate multiple candidate vaccines against a common placebo control, where new candidates can be added to the randomization as soon as they become available, meet local regulatory standards, and meet WHO’s prioritization criteria.^4^ These trials are rigorously designed with “virologically confirmed symptomatic COVID-19 symptomatic disease” as the primary endpoint. If “vaccine efficacy” denotes the relative reduction in the rate of such primary endpoint events in a vaccinated group of participants compared to placebo controls, then the WHO- and Food and Drug Administration (FDA)-recommended standard for worthwhile efficacy is having a point estimate of ≥50% vaccine efficacy with a 95% confidence interval (CI) lower bound of ≥30% efficacy, chosen to assure that deployed vaccines do more good than harm.^5,6^

Recently, initial reports of high efficacy in the short term for several vaccines have been published. Two vaccines manufactured by Pfizer/BioNtech and Moderna, which use novel mRNA technology, are yielding estimated efficacy of 94%–95%^7,8^ over a median follow-up of about 2 months. Additional reports of two vaccines that use Adenovirus vectors also have been disclosed, from AstraZeneca/Oxford in the United Kingdom and the Gamaleya Research Institute in Russia, with initial estimates of efficacy ranging from 62%–92%.^9,10^

The mRNA-based vaccines have started to become available, and access is expected to increase in many wealthy nations over the next several months. However, these vaccines have significant challenges in manufacturing and distribution, with requirements for cold temperatures during transport and storage that may make them particularly challenging for worldwide distribution in the short term. The adenovirus vaccines have potential advantages over the mRNA-based vaccines in manufacturability and distribution; with some countries including India building capacity to manufacture their own supply in the near future, the AstraZeneca vaccine may become available to a wide set of countries before the mRNA vaccines can.

However, even with these exciting reports, it is still important that other vaccine regimens be evaluated. Widespread implementation of multiple safe and effective vaccines will be needed given the breadth of the pandemic. Vaccines that can be administered in a single dose would be particularly useful in mass vaccination campaigns, and open questions remain about the durability of any vaccine effects and the potential for emerging concerns about safety.

## Methods

Non-inferiority studies can be an important tool in evaluating the efficacy of a new vaccine when there is one established in that population, and when randomization to placebo is not possible. The frequent goal in a non-inferiority trial is to reliably assess whether the efficacy of an experimental vaccine is not unacceptably worse than that of an active control vaccine that previously had been established to be effective, likely in a placebo-controlled trial. This is formally achieved by identifying a minimum threshold for what would constitute an unacceptable loss of efficacy, that is, a non-inferiority margin, and then designing the non-inferiority trial to rule out that margin. An important consideration in the design and conduct of non-inferiority trials is the need to address the inherent uncertainty about whether the effect of the active comparator vaccine, as estimated in its placebo-controlled trial, reliably represents its true effect in the setting of the non-inferiority trial. This is referred to as the constancy assumption.

To illustrate the fundamental importance of the constancy assumption, suppose an active comparator vaccine truly has vaccine efficacy of 95% over a short 2-month duration of follow-up, and true vaccine efficacy of 88% over 6 months of follow-up. Suppose further that, in its placebo-controlled trial, the active control vaccine was evaluated over only 2 months, but the non-inferiority trial will follow for events over 6 months. If it was inaccurately assumed that the active comparator vaccine would have the same 95% vaccine efficacy over 6 months, the resulting violation of the constancy assumption would lead to meaningfully overestimating an experimental vaccine with true 30% vaccine efficacy as being 71% = 100 [1 – (1 − 0.3){(1 − 0.95)/(1 − 0.88)}]%.

Based on these insights, one important consideration in the identification of the margin in the non-inferiority trial is to address the inherent uncertainty about the validity of the constancy assumption, while a second relates to ensuring the experimental vaccine achieves proper preservation of effect. These two are formally stated to be as follows:^11^

Consideration A: the non-inferiority margin should be formulated using adjustments to account for bias or lack of reliability in the estimate of the effect of the active comparator regimen in the setting of the non-inferiority trial.
Consideration B: the non-inferiority margin should be formulated to achieve preservation of an appropriate percentage of the effect of the active comparator regimen.

One could take several approaches to properly address Considerations A and B when formulating margins in non-inferiority trials, especially in the specific context of vaccines to stop a pandemic. A widely implemented approach with precedent for regulatory support is the “95–95” method,^11,12,13^ in which Consideration A is addressed by assuming the true effect of the active comparator vaccine in the non-inferiority trial would be the lower limit of the 95% CI for its estimated vaccine efficacy in the setting of the previously conducted randomized placebo-controlled trial(s); Consideration B is often addressed by preserving at least 50% of the effect of the active comparator vaccine, where, as discussed below, this effect is estimated using the active comparator to placebo hazard ratio (HR) in this time-to-event analysis setting.

Cox regression analyses are used to estimate the HR or relative rates of primary endpoint events on a vaccine versus a comparator regimen. Data from the placebo-controlled randomized trial that established the active comparator vaccine as having worthwhile efficacy are used to estimate the active comparator to placebo HR and, in turn, the active comparator’s vaccine efficacy is estimated as 100 (1 − HR). Working in the context of the estimated HR and thus, using the log-scale when calculating half the estimated effect, we are led to the following formula^12^ for the non-inferiority margin **δ**:

(1) $$\delta ={\left\{\frac{\left(95\%\mathrm{CI}\;\mathrm{upper}\;\mathrm{limit}\;\mathrm{of}\;\mathrm{the}\;\mathrm{HR}\right)}{{\left(95\%\mathrm{CI}\;\mathrm{upper}\;\mathrm{limit}\;\mathrm{of}\;\mathrm{the}\;\mathrm{HR}\right)}^{½}}\right\}}^{-1}$$

The term, (95% CI upper limit of the HR)^½^, is the “preservation of effect” adjustment and addresses Consideration B. Note that equation (1) simplifies to **δ** = (95% CI upper limit of the HR)^−½^, but we leave it in the expanded form in order to parallel proposed alternate margins below.

If the trial is conducted in a setting where there would be emerging availability of an effective vaccine and thus that would be a proper control regimen, yet at a time when current availability of safe and effective vaccines would not meet local and worldwide needs, then a non-inferiority margin more lenient than **δ** in equation (1) might be justified. Specifically, it may be sufficient that the strength of evidence of efficacy of the new vaccine in the non-inferiority trial would be equivalent to the strength of evidence meeting the WHO–FDA criteria for success in a placebo-controlled trial. In essence, the justification for using a weaker criterion is the recognition that multiple vaccines that are safe and have worthwhile efficacy are needed, even if a new vaccine might be less effective than one or more marketed vaccines previously established to have worthwhile efficacy. Since the WHO–FDA criteria for success, in part, requires evidence ruling out that the active control’s vaccine efficacy is ≤30%, this corresponds to the 95% CI upper limit of the active comparator to placebo HR being ≤0.70; in turn, the preservation of effect adjustment for a vaccine with efficacy at the threshold of achieving that criterion would be (0.70)^½^. This leads to the formula for the alternative non-inferiority margin:

(2) $$\delta_{o}={\left\{\frac{\left(95\%\mathrm{CI}\;\mathrm{upper}\;\mathrm{limit}\;\mathrm{of}\;\mathrm{the}\;\mathrm{hazard}\;\mathrm{ratio}\right)}{{\left(0.70\right)}^{1/2}}\right\}}^{-1}$$

Hence, in the non-inferiority trial, ruling out that the experimental to active comparator HR is ≥**δ_o_** allows the conclusion that the experimental vaccine is “at least similarly effective to” an active comparator vaccine having efficacy at the threshold for satisfying the WHO–FDA criteria for success, while ruling out that that HR is ≥**δ** allows the stronger conclusion that the experimental vaccine is “at least similarly effective to” the active comparator vaccine in the non-inferiority trial.

Finally, there might be reasons to choose a margin between **δ_o_** and **δ**, or one bounded by a specified maximally acceptable relative increase. For instance, there might be consensus among stakeholders that a non-inferiority margin could be no greater than 3 or 4; hence, ruling out that the experimental vaccine could have triple or quadruple the rate of symptomatic infections compared to an existing vaccine, regardless of the actual efficacy of existing vaccine. For illustration, a margin of min(**δ**,3) or, say, min(**δ_o_**,4) could be applied.

## Results

We will explore the properties of non-inferiority trials using margins **δ** and **δ_o_** in the context of the current state of COVID-19 vaccine research and development, balancing the feasibility of accruing large sample sizes or long durations of follow-up with appropriate rigor to identify vaccines that are reliably established to be meaningfully effective. We discuss the choice of margin as dependent on the efficacy of the available comparator vaccine in the country where the non-inferiority trial will be conducted. Two scenarios are considered in which an available active control vaccine has reliably estimated efficacy in preventing disease: first where vaccine efficacy is 90%–95% during the duration of the non-inferiority trial, and second where vaccine efficacy is 60% during trial duration. These two scenarios correspond to possible situations in countries where the mRNA vaccines or adenovirus-vectored vaccines become available, respectively, assuming their early estimates of vaccine efficacy will hold over longer-term follow-up. Calculations for additional scenarios are included in Tables 1–3.

**Table 1.** Consider non-inferiority trials designed with primary analysis to rule out the non-inferiority margin, **δ**, assuming vaccine efficacy of the experimental (EXP) and active control (AC) vaccines is equal.

From placebo-controlled RCT				For the non-inferiority trial					
Number events	Vaccine efficacy	Hazardratio	HR upper limit 95% CI	NI margin **δ**	NI margin **δ_o_**	H_A_: EXP/PLAhazard ratio	NI trial# events*	Maximum EXP/AC hazardratio ruling out	
								**δ** **	**δ_o_** **
175	95%	0.05	0.0855	3.421	9.790	0.05	34	1.631	3.795
350	95%	0.05	0.0730	3.700	11.454	0.05	31	1.686	4.038
175	90%	0.10	0.1525	2.561	5.486	0.10	54	1.456	2.880
350	90%	0.10	0.1348	2.724	6.207	0.10	48	1.490	3.071
175	80%	0.20	0.2845	1.875	2.940	0.20	112	1.282	1.972
350	80%	0.20	0.2566	1.974	3.260	0.20	97	1.310	2.110
175	70%	0.30	0.4162	1.550	2.010	0.30	225	1.189	1.535
350	70%	0.30	0.3781	1.626	2.213	0.30	184	1.212	1.638
175	60%	0.40	0.5480	1.351	1.527	0.40	470	1.126	1.271
350	60%	0.40	0.4997	1.415	1.674	0.40	355	1.147	1.354

RCT: randomized controlled trial; CI: confidence interval; HR: hazard ratio; NI: non-inferiority; PLA: placebo.

* Calculated assuming 90% power when vaccine efficacy of the experimental (EXP) and active control (AC) vaccines is equal, using a statistic having 2.5% false positive error when **δ** is the true EXP/AC hazard ratio.

** This represents the highest estimated experimental (EXP) to active control (AC) estimated hazard ratio that yields a positive result in the non-inferiority trial.

**Table 2.** Consider non-inferiority trials designed with primary analysis to rule out the non-inferiority margin, **δ_o_**, assuming that the experimental vaccine (EXP) would have 60% vaccine efficacy versus placebo.

From placebo-controlled RCT				For the non-inferiority trial					
Number events	Vaccine efficacy	Hazard ratio	HR upper limit 95% CI	NI margin **δ**	NI margin **δ_o_**	H_A_: EXP/PLAhazard ratio	NI trial# events*	Max EXP/AC hazard ratio ruling out	
								**δ** **	**δ**_o_ **
150	70%	0.30	0.4272	1.530	1.958	0.40	304	1.217	1.552
350	70%	0.30	0.3781	1.626	2.213	0.40	180	1.207	1.630
150	60%	0.40	0.5620	1.334	1.489	0.40	271	1.050	1.170
350	60%	0.40	0.4997	1.415	1.674	0.40	164	1.039	1.226
150	50%	0.50	0.6972	1.198	1.200	0.40	259	0.938	0.939
350	50%	0.50	0.6216	1.268	1.346	0.40	158	0.926	0.983

RCT: randomized controlled trial; CI: confidence interval; HR: hazard ratio; NI: non-inferiority; PLA: placebo.

* Calculated assuming 90% power when the experimental vaccine (EXP) has 60% vaccine efficacy, using a statistic having 2.5% false positive error when **δ_o_** is the true EXP/AC hazard ratio.

** This represents the highest estimated experimental (EXP) to active control (AC) estimated hazard ratio that yields a positive result in the non-inferiority trial.

**Table 3.** Consider non-inferiority trials designed with primary analysis to rule out the non-inferiority margin, **δ**, under the assumption that efficacy of the experimental (EXP) and active control (AC) vaccines is equal. While such trials properly are powered to rule out **δ** only when vaccine efficacy of the EXP vaccine truly is greater than or equal to that of the active control (AC), the trial is powered to rule out the non-inferiority margin, **δ_o_**, when the vaccine efficacy of the experimental (EXP) is only 10% less than that on the active control (AC). Results are presented corresponding to 175 or 350 events in the placebo-controlled trial of the AC.

Events in placebo-controlledtrial of AC	Vaccineefficacy of AC	NI trial #events	H_0_: true HR = margin		H_A_:		Power under H_A_	
			**δ**	**δ_o_**	EXP vaccineefficacy	HR	To ruleout **δ**	To ruleout **δ_o_**
175	95%	34	3.421	9.790	95%	1	90%	>99%
					90%	2	28%	90%
					80%	4	<1%	40%
	90%	54	2.561	5.486	90%	1	90%	>99%
					80%	2	13%	85%
					70%	3	<1%	41%
	80%	112	1.875	2.940	90%	0.5	>99%	>99%
					80%	1	90%	>99%
					70%	1.5	20%	90%
					60%	2	<1%	45%
350	95%	31	3.700	11.454	95%	1	90%	>99%
					90%	2	22%	90%
					80%	4	<1%	46%
	90%	48	2.724	6.207	90%	1	90%	>99%
					80%	2	16%	87%
					70%	3	<1%	48%
	80%	97	1.974	3.260	90%	0.5	>99%	>99%
					80%	1	90%	>99%
					70%	1.5	25%	93%
					60%	2	<1%	57%

HR: hazard ratio; NI: non-inferiority.

The approaches to non-inferiority presented in this article could also be contemplated as part of a hybrid approach, for settings where the placebo control is replaced by an active comparator vaccine, either in the same or a different trial. The hybrid approach would efficiently aggregate evidence about the efficacy of a candidate experimental vaccine, by combining evidence about the efficacy of that experimental vaccine obtained from the placebo-controlled and active comparator settings. Such hybrid approaches are not further considered here.

### Scenario 1

Scenario 1 involves the use of an active comparator vaccine having vaccine efficacy that is 95% over 2 months and 90% over 6 months, in non-inferiority trials designed with primary analysis to rule out the non-inferiority margin, **δ**.

The first scenario we consider is the one where a vaccine with very high efficacy becomes available in a region, such as the United States. For illustration purposes, we assume that this vaccine has been estimated to have 95% vaccine efficacy over 2 months and 90% over 6 months. While we consider both timeframes for a potential non-inferiority trial, we note that the number of participants and time to accrue an adequate number of infections with two highly effective vaccines make a 6-month non-inferiority trial more likely.

Considering first a 2-month trial, we assume an active comparator vaccine has estimated 95% vaccine efficacy, and a lower bound for the 95% CI of 0.9145 from 175 events in the placebo-controlled randomized trial. This level of evidence was achieved by both Moderna and Pfizer at the time of their requests to the FDA to grant an Emergency Use Authorization.^7,8^

Translating this onto the HR scale (see Table 1), the estimated active comparator to placebo HR is 0.05, with a 95% CI upper limit of 0.0855. Applying equations (1) and (2), we calculate the margins **δ** = 3.421 and **δ_o_** = 9.790. To have 90% power to rule out **δ** = 3.421, when preserving a 2.5% false positive error rate, a non-inferiority trial would be required to have 34 primary endpoints. The least favorable result to rule out the **δ** = 3.421 would be an estimated experimental to active control vaccine HR of 1.631, corresponding approximately to 21 versus 13 events on experimental to active control vaccines, respectively, allowing the conclusion that the experimental vaccine is “at least similarly effective to” the active comparator vaccine in the non-inferiority trial.

With 34 events, the least favorable result to rule out the **δ_o_** = 9.790 would be an estimated experimental to active control vaccine HR of 3.795, corresponding approximately to 26 versus 8 events on experimental to active control vaccines, respectively, allowing the conclusion that the experimental vaccine is “at least similarly effective to” an active comparator vaccine having efficacy at the threshold for satisfying the WHO–FDA criteria for success. As shown in Table 3, the experimental vaccine would need to have true vaccine efficacy of 95% for the trial would have high power to rule out **δ** and true vaccine efficacy of at least 90% for high power to rule out **δ_o_**.

Consider instead a 6-month trial comparing to an active comparator vaccine with 90% vaccine efficacy based on a randomized trial accruing 350 cases by this 6-month mark. In the HR scale, the estimated active comparator to placebo HR is 0.10, with a 95% CI upper limit of 0.1348. Applying equations (1) and (2), the margins are **δ** = 2.724 and **δ_o_** = 6.207. To have 90% power to rule out **δ**, when preserving a 2.5% false positive error rate, a non-inferiority trial would be required to have 48 primary endpoints (Table 1). The least favorable result to rule out the **δ** = 2.724 would be an estimated experimental to active comparator vaccine HR of 1.490, corresponding approximately to 28 versus 20 events on experimental to active comparator vaccines, respectively, allowing the conclusion that the experimental vaccine is “at least similarly effective to” the active comparator vaccine in the non-inferiority trial.

With 48 events, the least favorable result to rule out the **δ_o_** = 6.207 would be an estimated experimental to active comparator vaccine HR of 3.071, corresponding approximately to 36 versus 12 events on experimental to active control vaccines, respectively, allowing the conclusion that the experimental vaccine is “at least similarly effective to” an active comparator vaccine having efficacy at the threshold for satisfying the WHO–FDA criteria for success. As shown in Table 3, the experimental vaccine would need to have true vaccine efficacy of 90% for the trial to have high power to rule out **δ** and true vaccine efficacy of at least 80% for high power to rule out **δ_o_**.

In scenario 1, the 34-event trial comparing two vaccines having approximate 95% vaccine efficacy and the 48-event trial comparing two vaccines having approximate 90% efficacy would require approximately two- to three-fold person years of follow-up relative to a frequently used design of a 150-event placebo-controlled trial of a vaccine having 60% vaccine efficacy, assuming these trials were conducted in settings having similar attack rates. For this reason, as noted earlier, the scenario of the 6-month non-inferiority trial seems more likely.

Based on these insights, when the active comparator vaccine has very high efficacy, even when using **δ_o_**, the non-inferiority trial is unlikely to conclude that the experimental vaccines satisfy the WHO–FDA criteria for success unless they have true vaccine efficacy above 75%. While this is disappointing if the experimental would be a single dose vaccine with 65% efficacy, such insensitivity arguably is appropriate if the highly effective active control vaccine is readily available in a community, since randomization likely would be limited to an experimental vaccine hypothesized to have similarly high efficacy. In addition, this high bar helps protect against meaningfully overestimating the efficacy of an inadequately effective experimental vaccine when it is compared with an active control vaccine for which the efficacy has also been overestimated.

### Scenario 2

Scenario 2 involves the use of an active comparator vaccine having vaccine efficacy of 60% over 4–6 months, in non-inferiority trials designed with primary analysis to rule out the non-inferiority margin, **δ_o_**.

Suppose the placebo-controlled evidence for the active comparator vaccine exceeds the threshold for meeting the WHO–FDA criteria for success, by having 60% estimated vaccine efficacy and, with 350 events, a lower limit of the 95% CI that is 50.0%. Then, the estimated active comparator to placebo HR is 0.4 and the 95% CI upper limit of the HR is 0.500. Plugging in the observed upper bound of 0.500 into equations (1) and (2), **δ** would be 1.415 and **δ_o_** = 1.674 (see Table 2). This scenario might be close to what we could expect if the AstraZeneca/Oxford vaccine becomes available based on data similar to what has been described in initial reports.^9^

Continue to assume the placebo-controlled active comparator trial had 350 events and a new experimental vaccine has true vaccine efficacy of 60%. Then, the alternative hypothesis for the HR for the experimental to active control vaccine is 1.0. Under the hypothesis that the true HR is 1.0, and preserving a 2.5% false positive error rate, a non-inferiority trial based on a margin of **δ** = 1.415 would be required to have 355 primary endpoints for 90% power (see Table 1), whereas one based on **δ_o_** = 1.674 would require only 164 events (see Table 2). When using the wider margin **δ_o_**, the least favorable result to rule out that non-inferiority margin would be an estimated experimental to active comparator vaccine HR of 1.226, corresponding approximately to 90 versus 74 events on the experimental versus active comparator vaccines, respectively. When multiplied by the estimated vaccine efficacy for the active control vaccine of 0.40, this yields 0.486, corresponding indirectly to an inferred efficacy of the experimental vaccine to placebo of 51.4%.

As in scenario 1, the 164-event trial in scenario 2 would require approximately two- to three-fold person years of follow-up relative to a 150-event placebo-controlled trial of a vaccine having 60% vaccine efficacy, assuming these trials were conducted in settings having similar attack rates. However, unlike scenario 1, in scenario 2—where the active comparator vaccine has an estimated true vaccine efficacy in the range of approximately 60%, as detailed Table 2—trials would be well powered to rule out the non-inferiority margin, **δ_o_**, for any experimental vaccine having true efficacy of at least 60%, in turn justifying the conclusion that such vaccines would be “at least similarly effective to” an active comparator vaccine having efficacy at the threshold for satisfying the WHO–FDA criteria for success. Thus, conducting trials in scenario 2 would be an efficient and reliable approach for increasing available vaccines with “worthwhile” efficacy.

Further increases in efficiency could be obtained through interim monitoring. In scenario 2 where the active comparator vaccine would have vaccine efficacy of 60% over 4–6 months, for an experimental vaccine having considerably higher true efficacy, an interim analysis in the non-inferiority trial could be definitively positive. These interim evaluations could be achieved, for example, using standard group sequential monitoring boundaries to assess whether interim data are sufficiently favorable to rule out the non-inferiority margin **δ**. By implementing this approach recently in the HIV Prevention Trials Network #083 non-inferiority trial conducted in the setting of pre-exposure prophylaxis for HIV infection,^14^ early termination was justified when the experimental cabotegravir regimen had an estimated 66% relative reduction (HR 0.34, 95% CI 0.18, 0.62) in risk of HIV infection against the emtricitabine/tenofovir active comparator regimen.

## Conclusion

Non-inferiority trials using margins proposed in this article may provide the ability to obtain reliable randomized evaluations of efficacy and safety of experimental COVID-19 vaccines. Such trials are well powered to reliably evaluate experimental vaccines that truly are similarly effective to an active comparator vaccine having any level of “worthwhile” efficacy. However, when the active comparator vaccine has efficacy ≥90%, an important limitation of this non-inferiority approach is its low power to confirm, as worthwhile, a safe and effective experimental vaccine having a favorable 60%–70% level of efficacy and a desirable profile such as characteristics readily enabling mass production. Use of the proposed more lenient non-inferiority margin, **δ_o_**, would provide sensitivity to confirming the benefit of such an experimental vaccine when the active comparator vaccine has efficacy in the range of 50%–80%.

Non-inferiority trials, as presented in the scenarios in Tables 1 and 2, often require approximately two- to three-fold the person-years follow-up relative to a placebo-controlled trial of an experimental vaccine having hypothesized 60% vaccine efficacy. Given this, together with the likelihood that attack rates might be reduced by the impact of available vaccines with “worthwhile efficacy” in the regions in which the non-inferiority trial would be conducted, it seems likely that the duration of the non-inferiority trial would be 4–6 months, if not longer. In turn, to properly derive the non-inferiority margin, evidence about the effect of the active comparator regimen would need to be available over a similar duration.

The reliability of non-inferiority trials depends on the validity of the constancy assumption, that is, that the true efficacy of the active comparator vaccine in the setting of the non-inferiority trial will be accurately estimated using evidence about its effect from its placebo-controlled trial. Hence, validity of the non-inferiority trial could be influenced by factors that might meaningfully alter the efficacy of the active comparator regimen, such as whether the non-inferiority trial and the placebo-controlled trial that evaluated the active comparator vaccine are conducted in populations with adequately similar strains of SARS-CoV-2 virus and, as noted above, have similar durations of follow-up. To illustrate how the constancy assumption could be violated in an impactful manner, consider a plausible scenario where an active comparator’s efficacy is very high over the 2-month interval it was evaluated in the placebo-controlled trial, yet meaningfully wanes during the next 4 months. In a non-inferiority trial following participants over 6 months, if its non-inferiority margin was derived under the false assumption that the 2-month level of efficacy of the active comparator was sustained over 6 months, this violation of the constancy assumption would result in a substantial overestimation of the efficacy of the experimental vaccine. Hence, in potential scenarios considered in this article, a fundamentally important assumption is the duration of follow-up in the non-inferiority trial does not exceed the follow-up duration in the placebo-controlled randomized clinical trial that evaluated the active comparator vaccine.

The above scenario also makes it clear that, even in placebo-controlled trials that produce short-term 95% vaccine efficacy,^7,8^ it is important to continue to follow participants in a blinded manner as long as possible. While recent publications have provided strong motivation to do so based on the importance of obtaining reliable insights about durability of efficacy, long-term safety and effects on severe disease,^5,6,15^ it is important to recognize that extending the length of blinded follow-up would have the additional positive consequence of improving our ability to use such vaccines as active comparators in non-inferiority trials.

Placebo-controlled trials are particularly efficient in providing reliable and interpretable evidence about efficacy and safety of COVID-19 vaccines. They would be a preferred design in settings where countries have limited or no access to licensed vaccines having worthwhile efficacy.^15^ However, in settings where placebo-controlled trials would no longer be possible due to emerging availability of safe and effective vaccines, non-inferiority trials would be ethically and scientifically appealing, given the need for multiple safe and effective vaccines. There is considerable need for new vaccines that not only have a particularly favorable safety profile or improved efficacy but also could be administered in a single dose, without cold chain constraints, and with scalability enhancing the ability to enable mass vaccination campaigns. It is likely that non-inferiority trial designs, such as those discussed in this article, soon will be needed to achieve these objectives and, in turn, to succeed in the battle against the COVID-19 pandemic.
